# Puerarin attenuates renal fibrosis by reducing oxidative stress induced-epithelial cell apoptosis via MAPK signal pathways *in vivo* and *in vitro*

**DOI:** 10.1080/0886022X.2017.1305409

**Published:** 2017-03-24

**Authors:** Xiangjun Zhou, Chen Bai, Xinbo Sun, Xiaoxin Gong, Yong Yang, Congbo Chen, Guang Shan, Qisheng Yao

**Affiliations:** a Department of Urology, Taihe Hospital, Hubei University of Medicine, Hubei, China;; b Department of General Surgery, Taihe Hospital, Hubei University of Medicine, Hubei, China;; c Department of Urology, Renmin Hospital of Wuhan University, Hubei, China

**Keywords:** Puerarin, renal fibrosis, tubular injury, oxidative stress, epithelial cell apoptosis

## Abstract

Puerarin (PR) is an isoflavonoid isolated from the root of the plant *Pueraria lobata* and has been widely used in traditional Chinese herbal medicine for the treatment of various diseases. Oxidative stress and epithelial cell apoptosis play important roles in the renal fibrotic process. The present study aimed to determine whether or not PR inhibits renal fibrosis by reducing oxidative stress induced-epithelial cell apoptosis. *In vivo*, unilateral ureteral obstruction (UUO) induced renal fibrosis, and epithelial cell apoptosis. A total of 24 mice were randomly assigned to four experimental groups: sham, UUO alone, UUO +50 mg/kg PR, and UUO +100 mg/kg PR. *In vitro*, 200 μM hydrogen peroxide (H_2_O_2_) induced epithelial cell apoptosis. The experiments were dived into four groups: control, H_2_O_2_ alone, H_2_O_2_+50 μM PR, and H_2_O_2_+100 μM PR. Tubular injury was measured in the renal cortex of the mice through periodic acid-Schiff (PAS) staining, and the extracellular matrix (ECM) was measured through Sirius red (SR), immunohistochemistry (IHC) staining, and Western blot. Renal epithelial cell apoptosis was measured through terminal deoxynucleotidyl transferase-mediated dUTP Nick-End labeling (TUNEL), flow cytometry (FCM), and Hoechst assays. The protein expression of NOX4, caspase3, ERK, P38, and JNK was assessed through Western blot. PAS staining showed that PR decreased renal tubular injury in UUO mice. SR and IHC staining demonstrated that PR decreased the accumulation of ECM. PR treatment significantly inhibited epithelial cell apoptosis according to the results of TUNEL, FCM, Hoechst, and Western blot. Furthermore, NOX4 increased in UUO mice and decreased with PR treatment. H_2_O_2_-derived oxidative stress activated epithelial apoptosis and mitogen-activated protein kinases (MAPK), and PR treatment significantly reversed it. These results suggest that PR treatment ameliorates renal fibrosis by inhibiting oxidative stress induced-epithelial cell apoptosis through MAPK signaling.

## Introduction

Renal tubulointerstitial fibrosis is a common pathway in progressive renal disease that leads to functional deterioration and eventual loss of renal function.^1^ Unilateral ureteral obstruction (UUO) is one of the main causes of renal fibrosis. Following UUO, mechanical stretch, ischemia, hypoxia, or oxidative stress result to renal tubular cell injury. As such, renal tubular epithelial cells may either undergo cell apoptosis or necrosis or undergo a phenotypic transformation and acquire mesenchymal characteristics.^2^ Increased epithelial apoptosis activates cellular infiltration and interstitial fibrosis, and inhibition of interstitial tubular cell apoptosis attenuates progression to fibrosis.^3^^,^^4^ Previous studies have demonstrated that oxidative stress increased in UUO mice, and oxidative stress production may induce the activation of certain proapoptotic proteins to promote apoptosis.^5^ Reactive oxygen species (ROS), including superoxide and hydrogen peroxide, induce the activation of proapoptotic proteins and influence cell signaling pathways.^6^^,^^7^ Moreover, ROS can activate mitogen-activated protein kinase (MAPK) pathway; this is an important pathological mechanism of epithelial apoptosis induced by oxidative stress.^8^ Thus, the inhibition of the MAPK pathway activation and ROS-induced apoptosis may be a potential target therapy for chronic kidney diseases (CKD).

Puerarin (PR) is extracted from the root of the kudzu plant and has been widely used in traditional Chinese herbal medicine for the treatment of various diseases, including cardiovascular, cerebral, and pulmonary injuries.^9–11^ Some scholars have found that PR has antifibrotic effect, particularly on hepatic fibrosis.^12^ However, whether or not PR has anti-injury and antifibrotic effect on renal fibrosis remains to be elucidated. In this study, we determined that PR treatment might attenuate renal tubulointerstitial fibrosis. Moreover, the underlying mechanisms involved in oxidative stress induced-epithelial cell apoptosis and MAPK pathway are also discussed.

## Materials and methods

### Animals and experimental design

Male C57 mice weighing 15–20 g were obtained from Wuhan University (Hubei, China). UUO was performed as described previously,^13^ and the left kidney and ureter were exposed through a flank incision. The left ureter was ligated with 4–0 silk. Finally, the wound was closed in layers. Sham groups underwent identical surgical procedures, but the left ureter was simply manipulated. Renal fibrosis was induced in mice with UUO. The 24 mices were randomly assigned into four groups: sham group, UUO group, UUO plus PR (50 mg/kg, 100 mg/kg) group. Mice from the PR groups were administered with PR by gastro gavage at daily doses of 50 and 100 mg/kg (PR dissolved in dimethyl sulfoxide). Mice were sacrificed on the seventh day subsequent to surgery, and the obstructed kidneys were harvested. A sample of the kidney was fixed in 4% buffered paraformaldehyde and was embedded in paraffin for histological studies. The remaining kidneys were snap-frozen in liquid nitrogen and stored at −80 °C for protein extraction.

### Agents and antibodies

The purified natural product PR was obtained from Shandong Fangming Pharmaceutical Co (Shandong, China). Anti-rabbit fibronectin antibodies (Abcam, Boston, MA); antibodies to caspase3, ERK, P38, JNK (Cell Signaling Technology Inc, Danvers, MA); NOX4 (Santa Cruz Biotechnology, Santa Cruz, CA). TUNEL Apoptosis Assay Kit and Annexin V-FITC Apoptosis Detection Kit (Beyotime Biotechnology, Shanghai, China). PAS and SR staining Kit (Guge Biological engineering Co., Wuhan, China)

### Cell culture and treatment

The proximal tubular cell line HK-2 (human kidney 2) was purchased from the American Type Culture Collection (Manassas, VA). The cells were cultured in Dulbecco’s modified Eagle’s medium (DMEM, Gibco, Waltham, MA) containing 10% fetal bovine serum at 37 °C in a humidified atmosphere containing 5% CO_2_. The cell confusion reached 70–80% and was treated with 200 μM H_2_O_2_ for 24 h with PR or without PR.

### Periodic acid-Schiff (PAS) staining

The tissue sections were deparaffinized in xylene, hydrated in graded ethanol, and then fixed in methyl Carnoy’s solution for 15 min, followed by washing with phosphate buffer saline (PBS). Sections were then incubated with 0.5% periodic acid for 15 min. The slides were washed thrice in PBS. Subsequently, sections were stained with Schiff’s reagent for 30 min. Cell nuclei were stained with alum hematoxylin for 5 min. Based on PAS staining, tubular injury was determined using Tubular Injury Scoring Scale. Scoring was done according to grading of tubular dilatation, epithelial simplification, and brush border loss. A score of 0 means normal; 1 means less than 25% of the cortex is involved; 2 means 25–50% of the cortex is involved; 3 means 50–75% of the cortex is involved; and 4 means more than 75% of the cortex is involved.

### Sirius red (SR) staining

The tissue sections were deparaffinized in xylene, hydrated in graded ethanol, and then fixed in methyl Carnoy’s solution for 15 min, followed by washing with PBS. Sections were then incubated with SR for 30 min, and the slides were washed thrice with PBS.

### Immunochemistry staining

The tissue sections were deparaffinized in xylene and hydrated in graded ethanol. The antigens were retrieved by heating in 100 °C for 15 min. Tissue sections were treated with 0.5% bovine serum albumin solution for 15 min at room temperature, followed by thrice washing with PBS. Sections were then incubated first in solutions containing fibronectin antibodies overnight at 4 °C and then with secondary antibodies for 1 h.

### Terminal deoxynucleotidyl transferase-mediated dUTP nick-end labeling (TUNEL) staining

Kidney sections were deparaffinized, and rehydrated sections were stained with hematoxylin and eosin. Apoptotic cells were stained with *in situ* terminal deoxynucleotidyl transferase-mediated dUTP Nick-End labeling method for 1 h.

### Flow cytometry (FCM)

Briefly, a small piece of renal cortex was taken, and then organize grinding single-cell suspension was prepared. The cells were collected, washed once with PBS, stained with annexin V/PI according to the instructions of the manufacturer, and then analyzed using flow cytometry and Mac Cycle software (Bike Ride, Boston, MA).

### Hoechst staining

Cells were cultured at 37 °C in a humidified atmosphere containing 5% CO_2_ and then treated with H_2_O_2_ and PR. Hoechst (1:500) were added to the cells, and the cells were then incubated at 37 °C for 10 min. Hoechst-stained apoptotic cells are brighter compared with normal cells.

### Western blot analysis

Kidney samples were sonicated and resuspended in 0.4 ml RIPA lysis buffer. The protein concentration was determined using a bicinchoninic acid protein assay kit, and 100 μg total proteins were loaded in each well. The kidney samples were separated through sodium dodecyl sulfate–polyacrylamide gel electrophoresis on 10% polyacrylamide gels. Gels were electroblotted onto a polyvinylidene difluoride membrane. Blots were incubated first with primary antibodies overnight at 4 °C and then with peroxidase-conjugated secondary antibodies for 60 min at room temperature. Bound antibodies were detected using an ECL advanced system. Band intensities were analyzed using the Quantity one software.

### Statistical analysis

Data are presented as the mean ± standard deviation. One-way analysis of variance and the Student–Newman–Keuls test were used for quantitative data analysis. *p* < .05 was considered to indicate a statistically significant difference.

## Results

### PR attenuated tubular injury in UUO kidney

**Figure 1.**
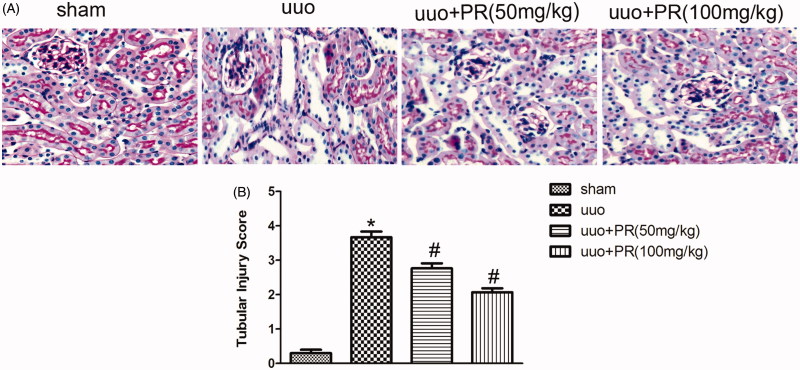
Effects of puerarin treatment in tubular injury after UUO in mouse. (A) Representative photomicrographs of periodic acid-Schiff staining of the four groups: sham, UUO, UUO +50 mg/kg PR, UUO +100 mg/kg PR. (B) Semiquantitative analysis of tubular injury. *p < .05 compared with the sham group; #*p* < .05 compared with the UUO group (magnification, ×200).

The effect of PR on the suppression of tubular injury was examined. PAS-stained micrographs of the kidney are shown in Figure 1. A significantly greater amount of tubular damage was observed in the UUO group compared with the sham group (*p* < .05); however, this damage was alleviated in the 50 mg/kg and 100 mg/kg PR group (*p* < .05).

### PR inhibited the accumulation of ECM in UUO kidney

**Figure 2.**
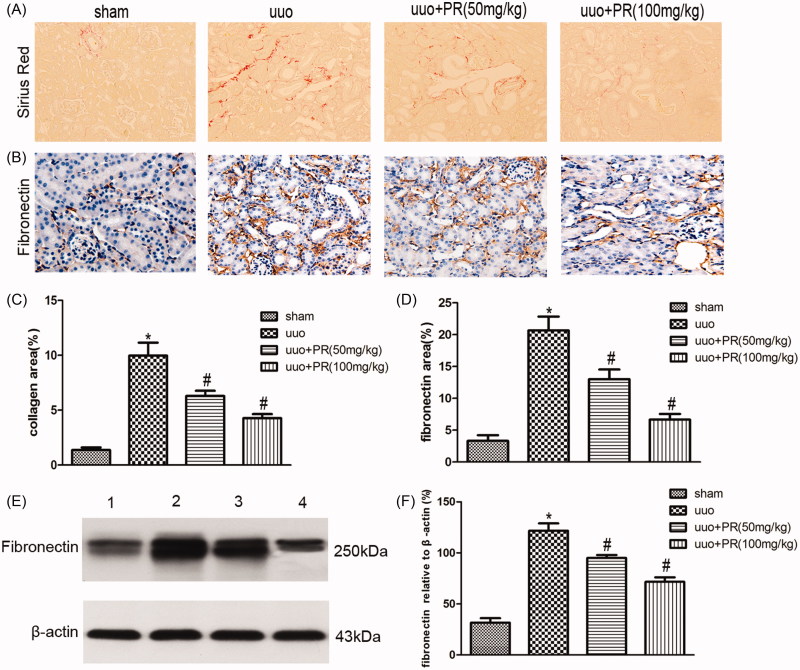
Effects of puerarin treatment in interstitial ECM after UUO in mouse. (A) Representative photomicrographs showing collagen area under Sirius red staining in the four groups: sham, UUO, UUO +50 mg/kg PR, and UUO +100 mg/kg PR. (B) Immunohistochemistry staining of fibronectin are shown in the four groups: sham, UUO, UUO +50 mg/kg PR, and UUO +100 mg/kg PR. (C and D) Semiquantitative analysis of interstitial collagen and fibronectin area. (E) Representative Western blots gels for fibronectin. Numbers 1, 2, 3, and 4 correspond to sham, UUO, UUO +50 mg/kg PR, and UUO +100 mg/kg PR groups, respectively. (F) Semi-quantitative analysis of the fibronectin area. *p < .05 compared with the sham group; #*p* < .05 compared with the UUO group (magnification, ×200).

The effect of PR on the suppression of accumulation of ECM was examined. SR staining of collagen, immunohistochemistry staining, and Western blot of fibronectin in the kidney are shown in Figure 2. There were significantly greater amount of ECM was observed in the UUO group compared with the sham group (*p* < .05); however, this fibrosis was alleviated in the 50 and 100 mg/kg PR treatment (*p* < .05).

### PR reduced oxidative stress in UUO kidney

**Figure 3.**
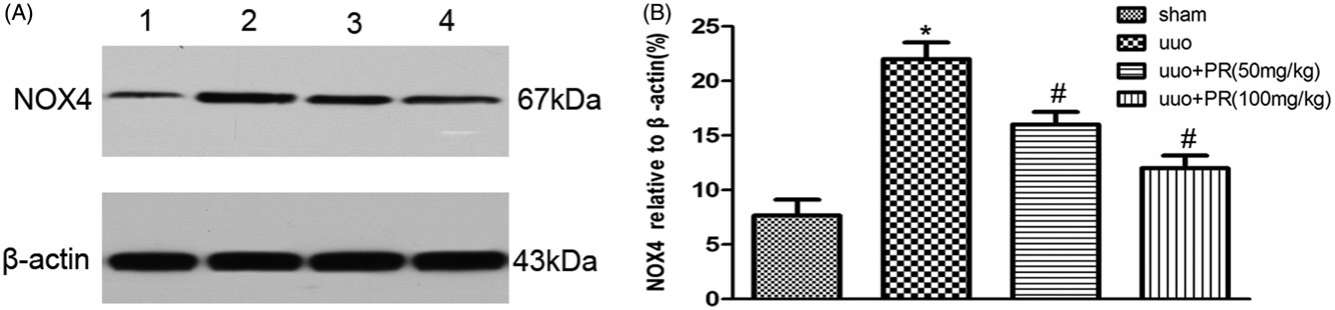
Effects of puerarin treatment in oxidative stress after UUO in mouse. (A) Representative Western blot gels for NOX4. Numbers 1, 2, 3, and 4 correspond to sham, UUO, UUO +50 mg/kg PR, and UUO +100 mg/kg PR groups, respectively. (B) Semiquantitative analysis of NOX4. *p < .05 compared with the sham group; #*p* < .05 compared with the UUO group.

The effect of PR on the reduction of oxidative stress was examined. Western blot of NOX4 in the kidney are shown in Figure 3. There were significantly higher level of NOX4 were observed in the UUO group compared with the sham group (*p* < .05); however, this level decreased in the 50 and 100 mg/kg PR treatment (*p* < .05).

### PR suppressed epithelial cell apoptosis in UUO kidney

**Figure 4.**
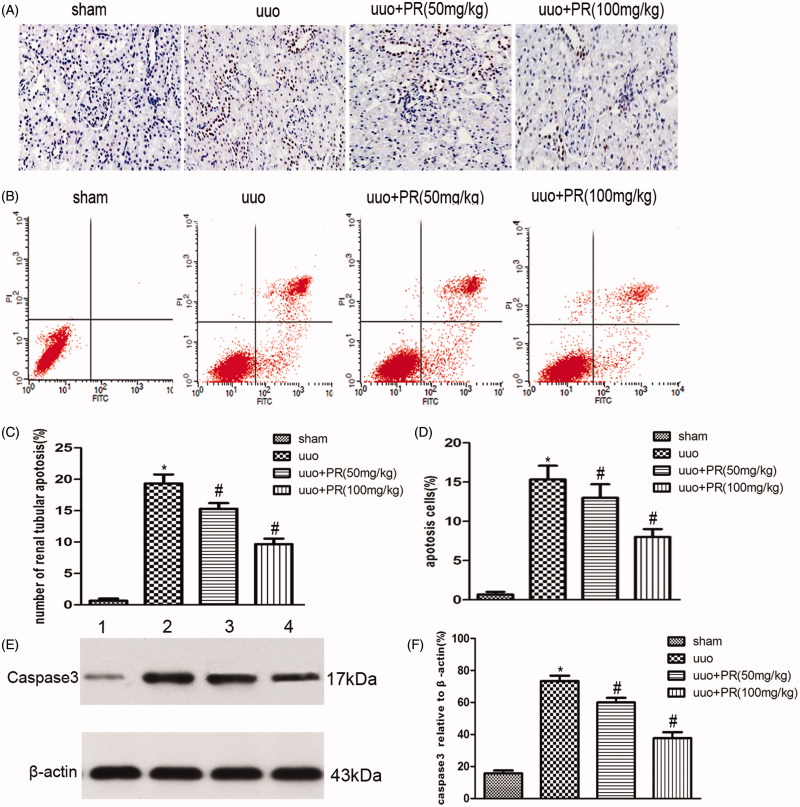
Effects of puerarin treatment in cell apoptosis after UUO in mouse. (A) Representative photomicrographs showing cell apoptosis in TUNEL staining in the four groups: sham, UUO, UUO +50 mg/kg PR, and UUO +100 mg/kg PR. (B) Representative photomicrographs showing cell apoptosis FCM in the four groups: sham, UUO, UUO +50 mg/kg PR, and UUO +100 mg/kg PR. (C and D) Semiquantitative analysis of apoptosis rate. (E) Representative Western blot gels for caspase-3. Numbers 1, 2, 3, and 4 correspond to sham, UUO, UUO +50 mg/kg PR, and UUO +100 mg/kg PR groups, respectively. (F) Semiquantitative analysis of caspase-3. *p < .05 compared with the sham group; #*p* < 0.05 compared with the UUO group.

Epithelial cell apoptosis, as identified by TUNEL assay and FCM, was prominent in the UUO kidney. This was substantially reduced by PR treatment. Western blot showed a significantly higher level of caspase-3 in the UUO group than the sham group, PR treatment dramatically reversed it in the 50 and 100 mg/kg (*p* < .05) (Figure 4).

### PR inhibited MAPK signaling pathway in UUO kidney

**Figure 5.**
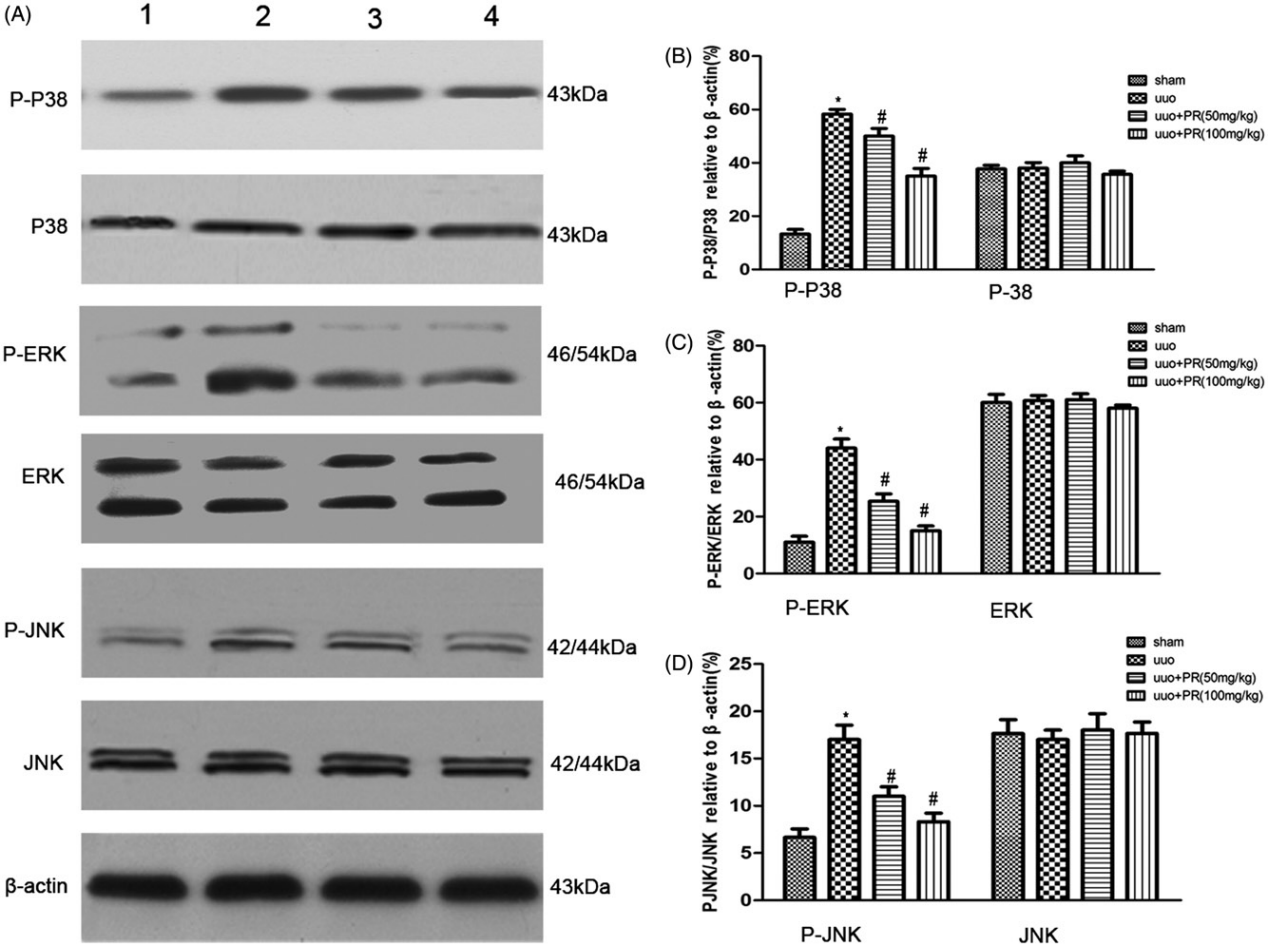
Effects of puerarin treatment in the expression of MAPK after UUO in mouse. (A) Representative Western blots gels for MAPK. Numbers 1, 2, 3, and 4 correspond to sham, UUO, UUO +50 mg/kg PR, and UUO +100 mg/kg PR groups, respectively. (B) Semiquantitative analysis of p38 and p-p38. (C) Semiquantitative analysis of ERK and p-ERK. (D) Semiquantitative analysis of JNK and p-JNK. *p* < .05 compared with the sham group; #*p* < .05 compared with the UUO group.

To investigate whether PR is involved in cell apoptosis through MAPK pathways, we examined levels of the MAPK protein in mice with UUO. Western blot analysis showed that PR treatment significantly decreased MAPK expression in mice with UUO (Figure 5).

### PR suppressed ROS-induced cell apoptosis in cultured renal tubular epithelia cells

**Figure 6.**
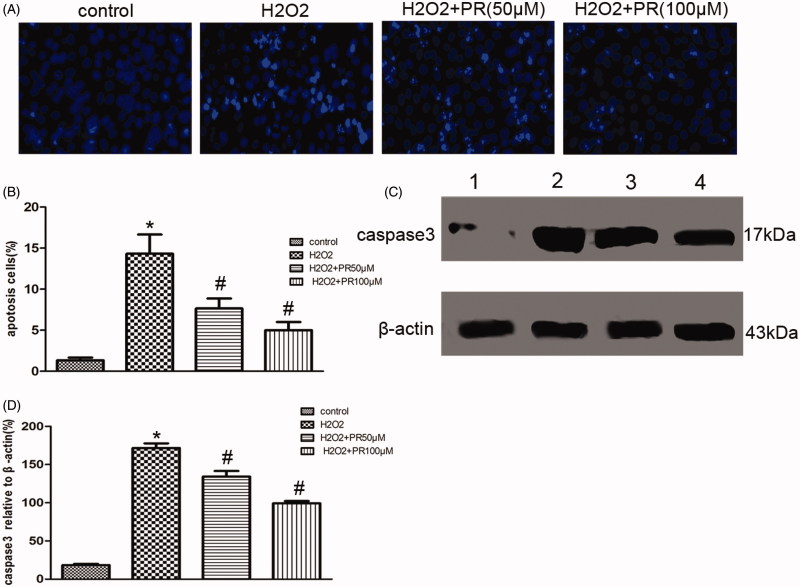
Effects of puerarin treatment in ROS-induced epithelial cell apoptosis. (A) Representative photomicrographs showing cell apoptosis in Hoechst staining in the four groups: control, H_2_O_2_, H_2_O_2_+50 μM PR, and H_2_O_2_+100 μM PR. (B) Semiquantitative analysis of apoptosis rate. (C) Representative Western blot gels for caspase-3.Numbers 1, 2, 3, and 4 correspond to control, H_2_O_2_, H_2_O_2_+50 μM PR, and H_2_O_2_+100 μM PR groups, respectively. (D) Semiquantitative analysis of caspase-3. *p < .05 compared with the control group; #*p* < 0.05 compared with the H_2_O_2_ group.

Hoechst staining revealed that ROS-induced epithelial cell apoptosis, PR treatment dramatically inhibited it. Western blot showed a significantly higher level of caspase-3 in the H_2_O_2_ group than the control group, PR treatment dramatically reversed it in the 50 and 100 μM (*p* < .05) (Figure 6).

### PR inhibited MAPK signaling pathway in cultured renal tubular epithelia cells

**Figure 7.**
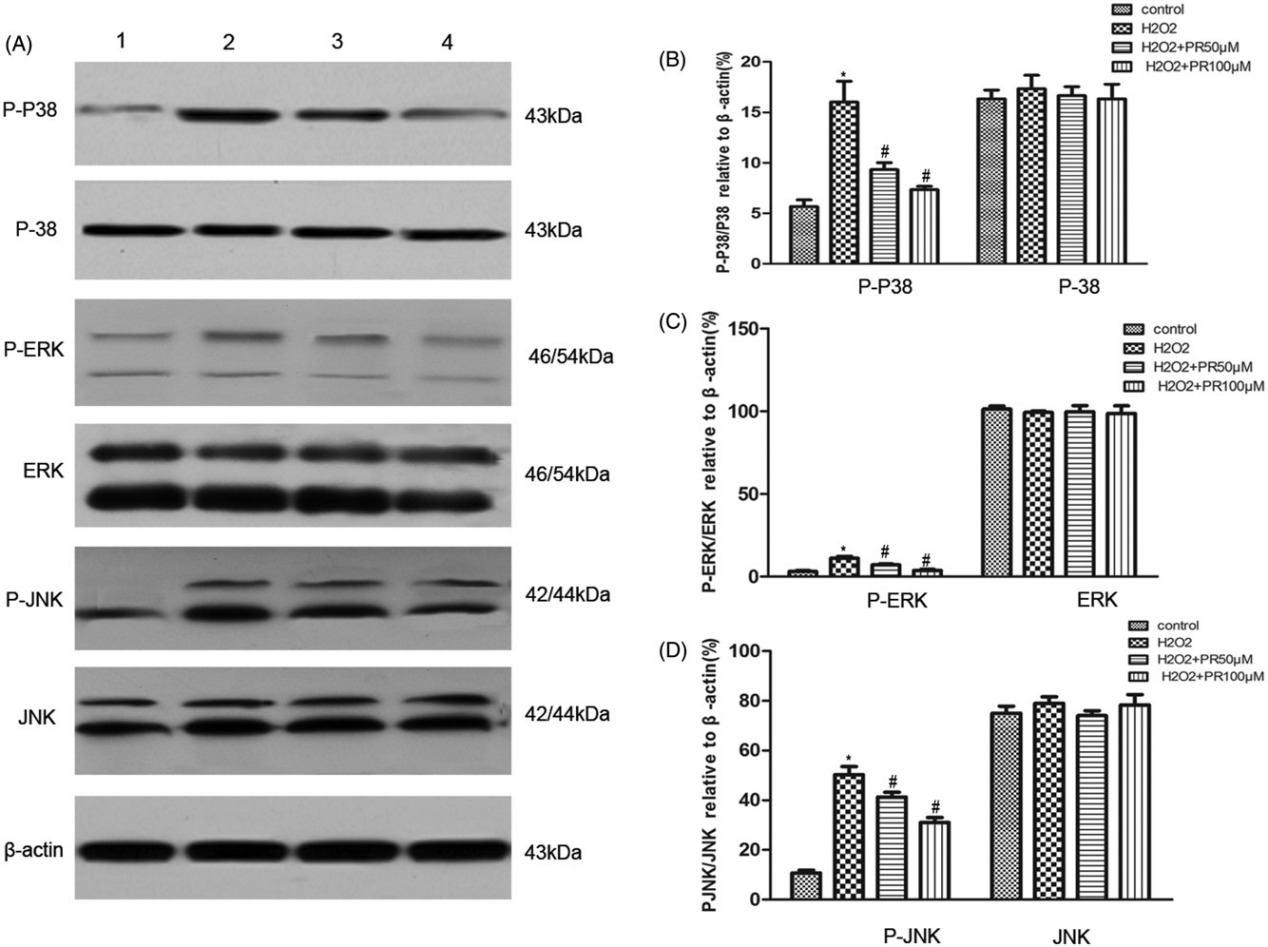
Effects of puerarin treatment on the expression of MAPK in epithelial cells. (A) Representative Western blot gels for MAPK. Numbers 1, 2, 3, and 4 correspond to the control, H_2_O_2_, H_2_O_2_+50 μM PR, and H_2_O_2_+100 μM PR groups, respectively. (B) Semiquantitative analysis of p38 and p-p38. (C) Semiquantitative analysis of ERK and p-ERK. (D) Semiquantitative analysis of JNK and p-JNK. *p < .05 compared with the control group; #*p* < .05 compared with the H_2_O_2_ group.

Western blot analysis data revealed that the expression of MAPK in the H_2_O_2_ group was more than that in the control group. Compared with the H_2_O_2_ group, the expression of MAPK in the PR treatment groups was significantly down regulated in the epithelia cells (Figure 7).

## Discussion

Fibrosis is ubiquitous among patients with CKD, regardless of the initial disease. It is characterized by the accumulation of pathological extracellular matrix (ECM) proteins in the interstitial space between nephrons. Fibrosis is strongly associated with and is a predictor of decline of kidney function.^14^ PR has been widely used in traditional Chinese herbal medicine for the treatment of various diseases. A previous study indicated that PR inhibited liver fibrosis induced by CCl4.^15^ Meanwhile, decreased ECM in the present study demonstrated that PR was able to attenuate renal fibrosis in mice with UUO.

Tubular epithelial cells are one of the primary targets in a variety of renal diseases. Renal tubular epithelial cells produce chemotactic factors through tubular stimulation, and cytokines, which actively participate in the occurrence and development of renal interstitial fibrosis, induce fibrosis phenotype into myofibroblasts and apoptosis. Therefore, intervention for renal tubular epithelial cell apoptosis may provide new insights and techniques for antifibrosis treatment. Following UUO, mechanical stretch, ischemia, hypoxia, and oxidative stress result to renal tubular cell injury. Depending on the severity and duration of the injury, tubular cells exhibit a wide range of responses.^16^^,^^17^ Oxidative stress plays a critical role in the initiation and progression of CKD; it may induce the activation of certain proapoptotic proteins.^18^ The apoptosis in renal tubular cells is involved in fibrosis after renal injury.^19^ Previous studies found that UUO induces the production of ROS, and increased ROS production triggers epithelial apoptosis.^5^ PR has exhibited an antioxidant effect in the treatment of liver diseases and Alzheimer’s disease. PR attenuated learning and memory impairments and inhibited oxidative stress in streptozotocin-induced Alzheimer’s disease.^20^ PR also effectively alleviated hepatic damage through potential antioxidant, anti-inflammatory, or antiapoptotic mechanisms.^21^ However, the effect of PR on renal fibrosis and oxidative stress-induced apoptosis remains unclear. Thus, the effect of PR was explored in our study. In this study, UUO significantly increased ROS production in vivo, and this has also been reported by previous studies.^5^ Importantly, PR significantly decreased ROS production and ROS-induced apoptosis *in vivo* and *in vitro*.

MAPKs, including ERK, JNK, and p38, are a family of serine/threonine kinases that may regulate cellular proliferation and apoptosis.^22^^,^^23^ Oxidative stress is known to activate MAPKs. A previous study demonstrated that various stress stimuli, including the oxidative stress caused by ROS, might induce potential activation of MAPK signaling pathways.^24^ In addition, MAPK activation contributes to stress-induced apoptosis.^25^ The results of the present study demonstrate that ROS induces phosphorylation of p38, ERK, and JNK. PR was also observed to inhibit MAPK activation, suggesting that PR attenuates renal fibrosis by reducing oxidative stress induced-epithelial cell apoptosis via MAPK signal pathways.

In conclusion, we provide evidence for the involvement of PR in the pathogenesis of renal tubulointerstitial fibrosis. This study may pave the way to prevent the progression of renal injury to CKD.
